# Implicit Representation of Grammatical Gender in Italian Children with Developmental Language Disorder: An Exploratory Study on Phonological and/or Syntactic Sensitivity

**DOI:** 10.1007/s10936-021-09788-x

**Published:** 2021-07-19

**Authors:** Caterina Artuso, Elena Fratini, Carmen Belacchi

**Affiliations:** Department of Communication Sciences, Humanities and International Studies (DISCUI), University of Urbino Carlo Bo, Via Saffi, 15, 61029 Urbino, Italy

**Keywords:** Developmental language disorder, Gendered language, Implicit language assessment, Semantic categorization, Forced choice categorization task

## Abstract

Children with Developmental Language Disorder (DLD) display impaired phonological and/or morpho-syntactic skills. To detect these impairments, it would be of value to devise tasks that assess specific markers of implicit linguistic competence. We administered a forced choice semantic categorization task developed in Italian (Belacchi and Cubelli in Journal of psycholinguistic research 41:295–310, 2012) for detecting the implicit use of grammatical gender markers in classifying epicenes names of animals: phonological and/or syntactic. Seventy Italian children with expressive-phonological DLD (mean age: 61.20 months) were compared with a same-size control group. Overall, the children with DLD performed more poorly than the control group. Also, the DLD participants used the phonological index to a significantly lesser extent, confirming their specific impairment in the phonological processing of words. The current study provided evidence for the status of phonological discrimination skills as a precursor of language development, and the value of using categorization tasks to assess implicit linguistic competence in children with DLD.

## Introduction

Children with Developmental Language Disorder (DLD) display limited linguistic competence despite being apparently typical in other respects (namely, at the cognitive, neurological, sensory and psychiatric levels), and despite receiving adequate social and educational opportunities for language learning (Bishop et al., 2016; Leonard, 1998). The clinical profile of children with DLD is heterogeneous; linguistic impairments can be present singularly or in combination, and some domains may be more compromised than others (Joanisse & Seidenberg, 1998; Rapin, 1996; Rapin & Allen, 1987).

Today, it is increasingly urgent to identify and administer tasks designed to detect markers of potential impairment and facilitate the diagnosis of DLD; advances in this regard will help compensate for impairment by facilitating timely intervention (see Capirci & Caselli, 2002). For example, Bortolini, Bonifacio, Fior, and Zmarich (1995) found that scarce or no canonical babbling at eight months of age usually predicts the development of DLD at later ages. This is particularly important given that DLD usually impacts negatively on children’s learning skills and school achievement (Rapin, 1996; Scarborough, 1990; Bishop, Snowling, & Stothard, 2000); in addition, DLD in pre-schoolers is often a predictor of learning disabilities at later ages (Scarborough, 1990).

In the linguistic phenotype of children with DLD, the morpho-syntactic domain tends to be more severely impaired than the phonological domain (Leonard, 2014). However, the two main vulnerability criteria are frequency of impairment and recovery times. With regard to frequency, impairment is most frequently observable in the expressive phonology domain, followed by the syntactic and lexical/morphological domains, and finally by comprehension, where impairment is less frequent. The opposite pattern holds for recovery, such that the most frequent forms of impairment are those most easily compensated for; hence, phonological impairments are usually better compensated for than syntactic ones (Bishop & Edmundson, 1987).

Phonological and morpho-syntactic impairments are usually assessed via verbal tasks using explicit verbal-linguistic stimuli such as words and non-words. For example, children with phonological impairments may display: poor ability to repeat words and non-words (Casalini et al., 2007; Dispaldro et al., 2013a; Gathercole & Baddeley, 1990); misarticulation or deletion of phonemes from words (Leonard, 1992; e.g., Italian children might say “*bitotto*” instead of “*biscotto*” [cookie]); difficulty in identifying words with similar phonemes (Bird & Bishop, 1992;), and poor phonological awareness (e.g., difficulty dividing a word into segments; Kamhi & Catts, 1986). Leonard and Eyer (1996) proposed that phonological difficulties may be the outcome of impairments in speech perception; indeed, impaired perception of speech could interfere with the development of phonological representations of words and this, in turn, could affect other aspects of language development such as morphology and syntax.

In addition, children with DLD may have difficulty producing and/or comprehending morphologically complex words such as past tense and plurals in English (e.g., *book-books*), case marking in Hebrew (Leonard & Eyer, 1996) and compound words in Greek (Dalalakis, 1999), or third person (singular/plural) subject-verb agreement in Italian (e.g., Bortolini et al., 1997). Accordingly, Rice and Wexler (1996) suggested that children with DLD are missing the abstract grammatical principle of inflection necessary to produce linguistic relationships such as grammatical case assignment and subject-verb agreement. Thus, errors in this domain may be the consequence of a lack of knowledge of morphological marking.

Again, children with DLD have also been found to display syntactic impairments in relation to complex structures/sentences (such as double-object alternations–e.g. in Italian saying “*Ho visto*” instead of “*L’ho visto*” [“*I saw*” versus the more appropriate form “*I saw it*”]), anaphoric reference, passives (see van der Lely & Harris, 1990; van der Lely & Stollwerck, 1997), and determiners (e.g., in Italian, saying “*Passami palla*” in place of the more appropriate form “*Passami la palla*” [“*Give me ball*” instead of “*Give me the ball*”]; Bortolini et al., 1997; Cipriani, Bottari, Chilosi & Pfanner, 1998). Specific comprehension impairments include difficulties in comprehending syntactic structures (Marini, Tavano & Fabbro, 2008) and grammatical components of language such as the determiner system (Bortolini et al., 1997; Cipriani et al., 1998; Clahsen, 1991; Fraser, Bellugi & Brown, 1963), as well as deficits in lexical-semantic organization (e.g., word-finding and vocabulary issues; Sheng & McGregor, 2010). A theoretically relevant debate in the field of general linguistics concerns the DP-NP gender agreement (see e.g., recent work from van Eynde, 2020); in our view, this is marginally relevant to our work, albeit in the current experimental task we have considered the role of the determiner (as syntactic marker) comparing it to a phonological marker (see next paragraphs).

### Clinical Markers of DLD in Italian

As stated above, the diagnosis of DLD in children has usually been based on tasks involving word recognition or production. More specifically, children’s scores on word and non-word repetition tasks (e.g., Casalini et al., 2007; Dispaldro et al., 2013a) have been identified as clinical markers in relation to language production. Such tasks have been successfully used across a range of languages including English (e.g., Chiat & Roy, 2007), Italian (e.g., Casalini et al., 2007), and Swedish (e.g., Sahlén et al., 1999). Italian-language versions of word and non-word repetition tasks have been found to display strong sensitivity and specificity (Dispaldro et al., 2013a, 2013b). In non-word and word repetition tasks, children listen to nonsense words or real words, respectively, and are then required to repeat them immediately. While non-word repetition mainly involves phonological abilities (Gathercole, 1995; Gathercole & Baddeley, 1990), the repetition of real words also requires semantic and morpho-syntactic abilities. Indeed, repeating words demands activation of their phonological form, as well as the retrieval of lexical/semantic knowledge from long-term memory (Casalini et al., 2007).

Other clinical markers have been identified. For instance, 7 years old children with DLD produce fewer *wh*- questions than typically developing children and this has been suggested as a measure to consider in DLD evaluation (Arosio & Guasti, 2019). In the same vein, children with DLD produce fewer third-person direct object clitics than same age peers, showing as a good clinical marker especially at ages of 5 and 7 years (Guasti et al., 2016). Children with DLD aged 7.6 years also have problems in comprehending relative clauses and in particular show more difficulties with object relative clause (rather than subject relative clause) that may be indeed considered a possible clinical marker too (Arosio et al., 2017). On the other hand, Contemori and Garraffa (2010) studied 4 preschool children with DLD analyzing their performance with relative clause finding a production deficit but not a comprehension one. More recently, Moscati and colleagues (2020) administered to children with DLD (aged 4–6 years) a forced choice task to study reception of syntactic configurations (e.g., subject-verb agreement) and found global weaknesses that increased as a function of complexity of the agreement configuration.

In sum, most of the studies were conducted on primary school children (rather than pre-schoolers) and critically, all the clinical markers (including both word and non-word repetition) require the repetition/comprehension/production of linguistic stimuli, and thus explicit knowledge and use of language, from children whose language and linguistic competence are clearly impaired. Given their focus on verbal language, these tasks may be perceived as extremely difficult and particularly unenjoyable by children with DLD. In general, children may not be motivated to engage with repetition tasks, leading them to perform particularly poorly and perhaps be mistakenly classified as displaying DLD (i.e., the nature of the tasks may generate a certain proportion of false positives in the diagnosis of DLD).

Furthermore, exclusive reliance on these measures (e.g., repetition tasks), which require explicit knowledge and use of language, may be not fully adequate for early assessment of DLD since they measure explicit language performance, thus picking up on the extent to which children are already manifesting the impairments they are being evaluated for. However, it would be of even greater interest to evaluate the implicit competence underpinning explicit language production (Flavell & Wohlwill, 1969).

For this reason, we believe that it would be of value to devise tasks for investigating implicit linguistic knowledge, understood as a prerequisite for explicit language competence. Learning about regularities, in both referential and formal-syntactic features of language, starts from implicit knowledge (e.g., Tsao et al., 2004); later, formal education fosters metacognitive knowledge and awareness of these patterns. In relation to the development of implicit knowledge and mastery of the phonological, syntactic and semantic features of language, a possible area of investigation is the formal representation of nouns’ grammatical gender. This is a particularly salient domain in gendered languages, that is to say, languages in which the grammatical gender of nouns is identified by phonological indexes (i.e., biased endings) and/or syntactic indexes (i.e., biased determiners).

### The Phonological and Syntactic Properties of Grammatical Gender

Although there is a well-established tradition of investigating the relationship between children’s semantic and syntactic abilities, mastery of grammatical gender and its sensitivity to different lexical markers has only recently become a research focus. Language learning is facilitated by language regularities; one of the most regular aspects in a gendered language is grammatical gender. Grammatical gender is an arbitrary categorization system that divides nouns into two (female and male, such as French, Italian, or Spanish) or more classes (e.g., three, such as German, or twenty such as Thai).

In Romance languages such as Italian, grammatical gender is expressed via suffixes. In general, grammatical gender is viewed as a system that is functionally independent of semantic and morphological information; in other words, it is independent of other formal and conceptual aspects of language. Indeed, the same concept may be referred to via nouns of different gender in different languages. For example, the same word may be feminine or masculine depending on the language (e.g., “*the sea*” is “*il mare*”, masculine, in Italian, but “*la mer*”, feminine, in French).

Nevertheless, gender classification within a given language can be quite regular, being based on salient semantic properties of referents (such as biological sex) and/or formal characteristics. Semantic regularities correlated with gender are often observed. For instance, in Italian, nouns designating plants are usually masculine, whereas nouns designating fruits are usually feminine; in contrast, in Latin, nouns designating plants are usually feminine, whereas nouns designating fruits are usually neutral.

Formal regularities may also be observed in both inflectional and derivational suffixes related to grammatical gender. In Italian (as well as in Spanish) masculine nouns usually end with the vowel -*o*, whereas feminine nouns end with -*a*. Also, the masculine determiner “*il/lo”* [the] usually identifies a masculine noun (e.g., *il* giorno, [the day]), whereas the feminine determiner “*la”* [the] usually identifies a feminine noun (e.g., *la* notte, [the night]; Bates et al., 1995).

It has consistently been shown that implicit knowledge of grammatical gender can influence children’s categorization abilities from pre-school age onwards. Children acquire the notion of biological gender at about 2.6 years (Fagot et al., 1986) and the ability to recognize the invariance of gender identity between 5 and 7 years (Wehren & DeLisi, 1983). Spanish children display the ability to use grammatical gender in a free classification task from the age of three (Martinez & Shartz, 1996). Flarty (2001) found that Spanish children (from the age of eight years), in a forced choice task where they had to name objects and then classify them as female or male, were influenced by the grammatical gender of the corresponding noun. Effects of grammatical gender have been observed in categorization tasks in French (Sera et al., 2002), Italian, and German (Agnoli & Forer, 2005); they occur earlier in languages characterized by shallow morphology–such as Italian and Spanish–than in English or French. Nevertheless, children learning a gendered language, such as Hebrew, are able to recognize their own and others’ gender identities earlier than those learning languages with no genders, such as English or Finnish (Beit‐Hallahmi, Guiora, Fried & Yoder, 1982).

A study by Seigneuric and colleagues (2007) investigated the effect of morpho-phonological markers of gender on classifying French pseudo-words ending with the masculine biased ending -*on*, or the feminine biased ending -*ette*, or the unbiased ending -*ique*. The authors showed that children from the age of three were sensitive to transparent gender markers and were able to appropriately associate a stimulus with a gendered determiner (i.e., *un* or *une*). In sum, research indicates that children make implicit use of grammatical gender for categorization purposes, but findings concerning the age at which they acquire this ability and the formal cues they rely on to apply it have been mixed.

More recently, Belacchi and Cubelli (2012) devised a semantic categorization task in which Italian children were asked to classify a set of photos of animals by their biological sex, on the basis of their bare names. Italian is a highly transparent language characterized by strong regularity in morphology, phonology and syntax. Therefore, it offers an appropriate medium for studying not only children’s representations of grammatical gender, but also, and most especially, their ability to implicitly process phonological and morpho-syntactic markers of gender; this in turn enables us to assess whether an Italian-speaking child has satisfactorily mastered the representation of grammatical noun gender, a property that serves to categorize words on a mainly arbitrary basis.

In the present study, children were shown 64 colored photos of animals in random order: half with feminine names and the other half with masculine names (see examples in Table 1). The participants were told that they would be shown photos of animals and then they were presented a forced choice task: if they thought an animal was male they were to place its photo in a blue box. On the contrary, if they thought an animal was female, they were to place its photo in a pink box. Importantly, the children were not requested to identify the grammatical gender of the animal names, but to classify as male or female the animals presented in photos. Responses were scored on the basis of categorization accuracy: a categorization was taken to be correct if in agreement with the grammatical gender of the animal noun.

**Table 1:** Examples of stimulus words

Grammatical gender index	Stimulus words examples
Referential. Nouns whose gender corresponds to the biological sex of the animal	(il) gallo [rooster, masculine]
	(la) gallina [hen, feminine]
Phonological-syntactic. Epicene with gender-biased ending (*a/o*) and beginning with a consonant (gendered determiner: *il/la* [*the*])	(*la*) farfall*a* [butterfly, feminine]
	(*il*) falc*o* [hawk, masculine]
Phonological. Epicene with gendered ending (*o/a)*, but beginning with a vowel (opaque determiner: *l’* [*the*])	(*l’*) aquil*a* [eagle, feminine]
	(*l’*) agnell*o* [lamb, masculine]
Syntactic. Epicene with the gender unbiased ending *e*, beginning with a consonant, (gendered determiner (*la/il*[*the*])	(*il*) piccion*e* [pigeon, masculine]
	(*la*) tigr*e* [tiger, feminine]

The 64 animal nouns presented in photos featured different combinations of linguistic–(i.e., phonological and syntactic)–markers of grammatical gender. The stimuli were divided into four groups of sixteen, based on these different combinations: (i) referential, i.e., nouns whose gender corresponds to the biological sex of the animal presented; (ii) phonological-syntactic, i.e., epicenes (that is nouns that can denote either the female or male animal) with gender-biased suffixes that begin with a consonant and thus require the gendered determiner *il/lo* for masculine, and *la* for feminine, nouns; (iii) phonological, i.e., epicenes ending with the masculine marker *o*, or the feminine marker *a*, but beginning with a vowel, thus requiring the determiner *l’* (the) which is opaque for gender; (iiii) syntactic, i.e., epicenes ending with the gender unbiased vowel *e,* but beginning with a consonant, and thus requiring the masculine determiner *il/lo* (the) or the feminine determiner *la* (the). See Table 1 for examples.

Belacchi and Cubelli (2012) found that preschool children (aged 3–5 years) were able to recognize word gender; more specifically, they showed that children in this age group were best at understanding referential markers (i.e., noun gender corresponding to the sex of the stimulus), followed by phonological-syntactic markers (i.e., biased ending + determiner), and phonological-only indexes (i.e., biased ending only). For these typically developing children, the most challenging type of gender marker to use was syntactic (i.e., determiner only).

In addition, the authors identified an interaction between the gender of the words to be categorized and the sex of the children participating in the study. They found that both girls and boys correctly classified a higher proportion of masculine items than feminine items, an effect also known as “male gender bias” (Boloh & Ibernon, 2010). In addition, the boys tended to be better at classifying the masculine items than the girls, whereas the girls displayed the opposite pattern, scoring a higher number of correct responses from feminine items than did the boys (“self-bias strategy”; Andonova, 2004; Mills, 2012).

Importantly, Belacchi and Cubelli’s (2012) findings could not be explained by semantic attributes of the target animals, such as size or wildness, which could provide clues to biological sex rather than by the grammatical gender of their noun labels. Indeed, the authors found that English-speaking adult participants accurately gender-classified animals whose biological sex is specified via their name (e.g., bull and cow) but performed at chance level in relation to animals whose Italian name is an epicene (see Experiment 2). Additional support to the patterns observed by Belacchi and Cubelli (2012) was found by Belacchi and Artuso (2020) who standardized the semantic categorization task for Italian pre-schoolers; the test named TGGI (test for implicit grammatical gender representation) showed good psychometric properties on a sample of 434 children aged 37–73 months. In the present study, we set out to verify whether the pattern of results obtained by Belacchi and Cubelli (2012) and Belacchi and Artuso (2020) with typically developing children would be replicated in children with DLD and whether the animal gender-classification task can usefully contribute to early screening for DLD in children.

### The Current Study

The aim of the current study is twofold. First, we set out to compare how children with DLD performed, vis-à-vis children in a control group, on the grammatical gender implicit semantic categorization task devised by Belacchi and Cubelli (2012) with a view to observing whether they displayed similar developmental patterns. More specifically, we wished to establish, in the case of children with DLD, which kind of gender markers facilitated them the most in categorizing the biological sex of animals: lexical-semantic markers (referential nouns), phonological-syntactic markers (epicenes with transparent endings + determiners), phonological markers (epicenes with transparent endings but gender-neutral determiners), or syntactic markers (epicenes with opaque endings but gendered determiners.

All these types of marker, whether phonological (e.g., Bird & Bishop, 1992), syntactic (the determiner system, e.g., Bortolini et al., 1997) or referential (i.e., reduced vocabulary use, e.g., Sheng & McGregor, 2010), are known to be particularly vulnerable in children with DLD. Overall, we expected the children with DLD to display poorer categorization abilities than the control participants, given the former group’s generalized delay in language development. In addition, we predicted that if the impairments of children with DLD fell mainly in the expressive-phonological domain, they would perform more poorly in the categorization of transparent-ending words beginning with a vowel (i.e., displaying lesser ability to use gendered-biased suffixes); on the other hand, if their impairment fell mainly in the syntactic domain, we expected them to perform more poorly in the categorization of epicenes with opaque endings beginning with a consonant (i.e., less competence in the use of determiners). We tested children aged 37 through 81 months, treating age as a continuous variable (i.e., a covariate, see "Method" section). Specifically, we predicted that there would be no age-related differences in performance between the two groups, because in children with DLD, language impairment has usually been found to be only marginally related to age (Leonard, 2014). In addition, we believe research on grammatical gender representation across languages is a timely and under investigated research topic that deserves attention (see the recent review by Samuel et al., 2019).

A secondary aim of our research was to control for the effects of male gender bias, and self-gender bias in children with DLD vis-à-vis the control group. We hypothesized that given their lesser mastery of language in general, children with DLD would also be less competent in differentiating between feminine and masculine in grammatical gender categorization, and therefore that the effects of the two biases in question would be less marked in the experimental group than in the control group.

## Method

### Participants

We recruited 70 Italian children who had been clinically diagnosed with DLD based on standard assessment criteria in the domains of overall cognitive development, phonological discrimination skills, lexical comprehension and production, syntactic comprehension. Data were collected from different Child Development Centers located in Central Italy. The diagnosis of DLD and the the subsequent official certification was released from the clinical group in charge of the child evaluation. We were allowed to access the diagnosis but not the clinical records (with all tests administered and scores). To establish the diagnosis, the child had to score at least -2SD in at least 2 linguistic standardized tests.

Also, we want to notice that the Italian Consensus Conference that established criteria for DLD diagnosis was held in 2019 and the current data were previoulsy collected (2015–2017). Therefore, before 2019, a standard clinical protocol was not yet established but a series of tasks should be done (and could differ) but always presented (i) a general cognitive skill measure (i.e., WISC IV, WIPPSI or LEITER), (ii) one or more expressive language test (i.e., TROG, fluency test, both semantic and phonemic), and (iii) one or more receptive language test (i.e., Peabody, PhonoLexical Test).

Most of these children had been diagnosed as displaying DLD in the expressive-phonological domain; indeed, they displayed deficits in the production of both phonological components (i.e., phonological processing) and syntactic components (i.e., use of articles, clitics, connectives) of language. Children with DLD showed neither neurological nor sensory impairments.

We compared the children with DLD with a control group of 70 children who had not been diagnosed with any cognitive or language impairment and were perfectly matched with the clinical DLD group in terms of age, gender and socio-cultural background, having been recruited from public early childhood education facilities in the same geographic area. See Table 2 for a breakdown of participants’ demographic characteristics. The study was conducted in accordance with the ethical standards laid down in the 1964 Declaration of Helsinki and fulfilled the standard ethical guidelines recommended by the Italian Association of Psychology (AIP). Written informed parental consent, as well as oral informed child assent, were obtained prior to participation, in keeping with the ethical standards of our university.

**Table 2:** Participants by age range, mean age (in months) and sex

	N	Age range	Mean age (*SD*)	Males
Children with DLD	70	37–81	61.20 (10.80)	50
Control group	70	37–81	61.21 (11.45)	50

### Materials and Procedure

We administered to both groups of participants the semantic categorization task that was originally devised by Belacchi and Cubelli (2012) and is described in detail in the Introduction to this paper. For examples of animal photos and for the complete list of animal nouns administered, see Belacchi and Cubelli (2012) and Belacchi and Artuso (2020). Photos were presented on a uniform white background.

The groups of stimuli were not matched for word frequency and familiarity, since nouns of referential group were more frequent and more familiar than the stimuli belonging to the other categories (i.e., the epicenes; see elementary lexicon Italian norms in Marconi et al., 1994). However, as the critical comparison is not between epicenes and referential, rather between different types of phonological and syntactic combinations, word frequency and familiarity are not considered critical variables (see also Belacchi & Cubelli, 2012).

### Procedure and Scoring

All children were tested individually in a quiet room at the child development center (for children with DLD) or at school (for the control group). The same experimenter tested both groups of children and was an undergraduate psychology student, not a member of the clinical staff who had diagnosed the children with DLD.

Children were shown the photos in random order and asked to name them; whether they had correctly named the animal or not, the experimenter next stated the bare name of the animal (i.e., the animal noun without any determiner). Thus, all participants were provided with the correct bare noun corresponding to each animal photo. Afterwards, the children were asked to categorize each animal as male or female by placing the photo in a blue or a pink box. More specifically, they were told that they would be shown photos of animals; and that if they thought that the animal was a male, they were to place the photo in a blue box; on the contrary, if they thought that the animal was a female, they were to place the photo in a pink box. Importantly, the children were not asked to identify the grammatical gender of the animal nouns, but to classify the animals presented in the photos according to their perceived biological sex (female or male).

At the beginning of the experimental session, three practice photos were used to familiarize the children with the procedure. The experimenter made sure that children knew the difference between male and female gender, and that they were able to grasp the color-coding of gender difference and apply it to animals.

The categorization response was classified as “correct” if congruent with the grammatical gender of the animal noun. A score of 1 was assigned for each answer that was correct (i.e., in agreement with the grammatical gender of the animal noun), and a score of 0 for each incorrect response. The possible range of scores was 0–64 for the overall task; and 0–16 for each of the four types of index. Latency of response was not taken into account, and “don’t know” responses were not admitted.

## Overview of Statistical Analyses

We used a mixed-effects approach to test our hypotheses; the most important advantage of mixed-effects models is that they allow all the factors that may contribute to understanding the data structure to be assessed simultaneously. These factors comprise not only the standard fixed-effect factors controlled by the experimenter but also random-effect factors, that is to say, factors whose levels are drawn at random from a population. In the model that best fitted our data, the fixed effects comprised Grammatical Gender Marker (referential, phonological-syntactic, phonological, syntactic), Actual Word Gender (female, male), Group (children with DLD, control group) and Sex of Participants (boys, girls). The random effects comprised the sample of children and Attributed Word Gender.

The children’s age was treated as a continuous variable and entered in the model as a covariate. Participants’ accuracy of gender categorization was treated as the dependent variable. Statistical analyses were performed using Rstudio software; for generalized mixed-effect models the R package lme4 was used; for planned comparisons the emmeans package was used, and the Tukey correction was used to check for Type I error.

## Results

The children’s age wielded no significant effect, *F* (1, 136.59) = 1.68, *p* = 0.20, and did not interact significantly with any other factors. This was in line with our hypotheses, confirming that no significant differences arise between the ages of 37 to 81 months, and that impairment (in our sample) was only marginally related to age.

The difference between the two Groups was significant, *F* (1, 135.04) = 88.45, *p* < 0.001. On average, children with DLD correctly categorized a lower number of animal nouns (*M* = 36.96 out of 64, *SD* = 5.58) than did their peers in the control group (*M* = 47.91 out of 64, *SD* = 6.45).

Sex of Participants also wielded a significant effect, *F* (1, 135.03) = 12.08, *p* < 0.001. On average, girls (*M* = 45.05 out of 64, *SD* = 7.66) categorized a higher number of animals than did boys (*M* = 41.46 out of 64, *SD* = 8.14).

Type of Grammatical Gender Marker also wielded a significant effect, *F* (3, 816.00) = 73.90, *p* < 0.001. Pairwise comparisons of the overall means showed that the participants were better at categorizing based on referential markers (*M* = 12.83 out of 16, *SD* = 0.20) as opposed to phonological-syntactic markers, *t*(816) = 12.40, *p* < 0.001 (*M* = 10.60 out of 16, *SD* = 0.20), phonological markers, *t*(816) = 13.33, *p* < 0.001 (*M* = 9.60 out of 16, *SD* = 0.21), *p* < 0.001 or syntactic markers, *t*(816) = 13.33, *p* < 0.001 (*M* = 9.41 out of 16, *SD* = 0.18), *p* < 0.001. Phonological-syntactic markers were used more effectively for categorization purposes than were phonological markers, *t*(816) = 4.02, *p* = 0.001 or syntactic markers, *t*(816) = 4.94, *p* < 0.001. Phonological markers and syntactic markers did not significantly differ in terms of the frequency with which they were used accurately, *t*(816) = 0.92, *p* = 0.80.

The actual Grammatical Gender of Words wielded a further significant effect, *F* (1, 136.00) = 61.99, *p* < 0.001. On average, male words were more accurately categorized (*M* = 24.21 out of 32, *SD* = 1.14) than female ones (*M* = 19.06 out of 32, *SD* = 1.20).

The interaction between Grammatical Gender marker and Group was significant, *F* (3, 816.00) = 6.05, *p* < 0.001. Planned comparisons were conducted for the two groups of participants separately.

**Fig. 1:**
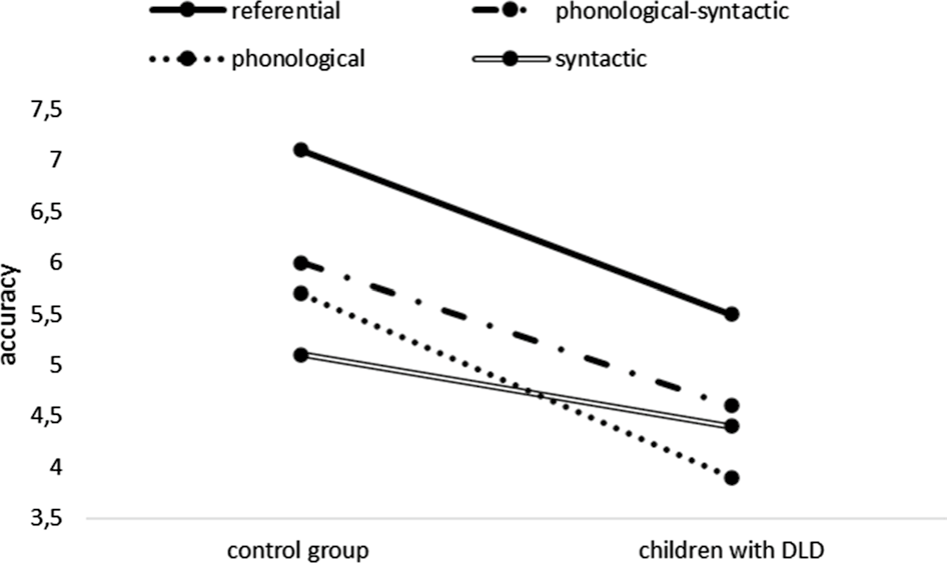
The effects of Grammatical Gender Index on participants’ accuracy of semantic categorization (for both control and DLD groups). The plotted dots represent mean accuracy values and the bars represent 95% CIs

In the control group, referential markers led to accurate categorization more frequently than any of the other type of marker: whether phonological-syntactic, *t*(816) = 7.18, *p* < 0.001; phonological, *t*(816) = 8.16, *p* < 0.001; or syntactic, *t*(816) = 11.44, *p* < 0.001. The phonological-syntactic and phonological indexes were associated with similar levels of categorization accuracy, *t*(816) = 0.99, *p* = 0.75. Both the phonological-syntactic index and the phonological index favored accurate categorization more than did the syntactic index: *t*(816) = 4.26, *p* < 0.001, and, *t*(816) = 3.27, *p* = 0.006, respectively. See Fig. 1.

In the children with DLD, the referential marker was associated with accurate categorization more frequently than were any of the other types of index: whether phonological-syntactic, *t*(816) = 4.64, *p* < 0.001; phonological, *t*(816) = 9.40, *p* < 0.001; or syntactic, *t*(816) = 7.34, *p* < 0.001. In addition, phonological-syntactic markers were associated with accurate categorization more frequently than either phonological, *t*(816) = 4.77, *p* < 0.001, or syntactic ones, *t*(816) = 2.71, *p* = 0.03. Finally, the phonological and syntactic markers were associated with similar levels of accuracy, *t*(816) = 2.05, *p* = 0.18.

As suggested by the main effect of Group reported above, children with DLD generally obtained lower scores than did their peers in the control group. However, the extent of this difference varied as a function of grammatical gender marker. Hence, we report the magnitude of the difference between the two groups (expressed as a *t* value) for each marker type: referential markers, *t*(574.43) = 7.50, *p* < 0.001; phonological-syntactic markers, *t*(574.43) = 5.09, *p* < 0.001; phonological markers, *t*(574.43) = 8.30, *p* < 0.001; and syntactic markers, *t*(574.43) = 3.62, *p* = 0.003. Importantly, phonological accuracy best discriminated between the groups, followed by referential accuracy, and syntactic accuracy. The least discriminating marker was phonological-syntactic accuracy.

This result confirms that the children with DLD (in our sample) were most compromised when required to process phonological aspects of language (in this case, by recalling and interpreting gender-biased suffixes); in addition, they displayed more limited lexical/semantic knowledge than their typically developing counterparts in the control group (see their lesser ability to accurately categorize using referential markers). When it was possible for them to draw on a syntactic marker (biased determiner) as well as a phonological marker (biased ending), their gender categorization became moderately more accurate.

**Fig. 2:**
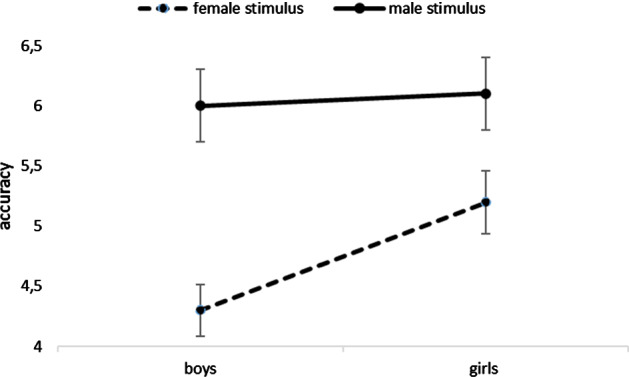
The effects of children’ s Sex on Assigned Word Gender. The plotted dots represent mean accuracy values and the bars represent 95% CIs

There was a significant interaction between actual Word Gender and Sex of participants, *F* (1, 136.00) = 5.67, *p* = 0.01. Both girls, *t*(136) = 3.21, *p* = 0.001, and boys, *t*(136.05) = 9.85, *p* < 0.001, were better at categorizing masculine words than feminine words, as shown in Fig. 2. While masculine words were equally accurately categorized by boys and girls [*t*(136.14) = 0.42, *p* = 0.67], the girls were more accurate at categorizing the feminine words than were the boys, *t*(136.25) = 3.87, *p* = 0.001.

## Discussion

In the current study, we administered a semantic categorization task to children with DLD. We introduced this measure of implicit language competence with a view to developing a tool for the early diagnosis of DLD (in pre-school children). Early screening is crucial given that DLD can have dramatic knock-on effects on learning skills and school achievement later in children’s school careers (e.g., Bishop et al., 2016; Rapin, 1996; Scarborough, 1990; Snowling, Bishop & Stothard, 2000). The tasks traditionally used to assess DLD are verbal (typically involving word and non-word repetition); however, in our view and as previously discussed, these tasks are likely to be perceived as difficult and unenjoyable by children with DLD. The fact that children are unlikely to feel motivated to engage with these tasks may lead them to be erroneously classified as having DLD. For this reason, it would be more suitable to devise tasks that indirectly measure implicit linguistic competence while bypassing explicit linguistic knowledge.

The task we used in the current study indirectly assesses linguistic skills such as, for instance, implicit competence in both the syntactic and the phonological features of language, thus providing insight into the development of grammatical gender representation. We specifically focused on the Italian language, a highly transparent language characterized by strong regularities in the domains of morphology, phonology and syntax; in Italian, the grammatical gender of nouns may be identified by phonological markers (i.e., gendered noun endings) and/or syntactic markers (i.e., gender-biased determiners). The task we administered had originally been devised by Belacchi and Cubelli (2012) to study grammatical gender representation in typically developing children and included different combinations of phonological and syntactic indexes: referential, phonological-syntactic, phonological and syntactic, as illustrated in Table 1 examples.

Belacchi and Cubelli (2012) found that preschool children with typical development were able to recognize the gender of words and mainly used referential markers (i.e., gender corresponding to the sex of the stimulus), followed by phonological-syntactic markers (i.e., biased ending + determiner) and phonological markers (i.e., biased ending only) to index gender. Syntactic gender indexing (i.e., based on the associated determiner only) was found to be the most challenging for this age group. Findings were confirmed by Belacchi and Artuso (2020) standardization study of the semantic categorization test for Italian pre-schoolers.

In the present study, we administered the animal sex classification task to a group of children with DLD and a control group with no linguistic impairment to observe whether the two groups displayed similar developmental patterns of different grammatical gender indexes. Previous research indicates that all modes of gender indexing, from phonological (e.g., Bird & Bishop, 1992) through syntactic (the determiner system, e.g., Bortolini et al., 1997) and referential (i.e., limited vocabulary use; Sheng & McGregor, 2010), are particularly vulnerable in children with DLD.

First, we identified a generalized delay in children with DLD (relative to the control group). Second, we observed a largely similar pattern of responses in the two groups, who performed comparably on the referential and phonological-syntactic items. The main difference between groups concerned the ability to use phonological markers of gender (a competence that is more impaired than syntactic indexing in children with DLD, whereas syntactic indexing is more difficult for typically developing control subjects). In other words, while the children in the control group were better at using phonological markers to categorize nouns by gender (as compared to syntactic markers, thus confirming the findings of Belacchi & Cubelli, 2012), the children with DLD made less accurate use of phonological markers of gender because phonological tasks are especially difficult for them.

Indeed, the phonological items appeared to discriminate the most effectively between children with and without DLD; the second-best discriminant was the set of referential items, followed by the syntactic items (see Fig. 2). The least discriminating items were those that relied on phonological-syntactic markers of gender (in which both types of formal marker, phonological and syntactic, are available). Thus, the findings suggest that the children with DLD in our sample displayed the strongest degree of impairment when it came to processing phonological aspects of language. They also displayed more restricted lexical/semantic knowledge than their typically developing counterparts (given that they found it more difficult to categorize the referential animal nouns by gender). On the other hand, the concurrent availability of both phonological and syntactic markers of gender (biased suffixes + biased determiner) made categorization moderately easier for them.

In addition, our data added support to sex differences in language development and in particular to its earlier development in girls over boys. Indeed, also in an implicit grammatical gender categorization task, girls showed better performance than boys did, regardless the specific marker considered (see also Bornstein et al., 2004; Ullman et al., 2008).

The current study lends support to findings already reported in the literature, especially in relation to phonological competence as a precursor of language development. For example, Tsao and colleagues (2004) demonstrated in a longitudinal study that, in typically-developing children, speech perception at 6 months of age (assessed via a standard measure of the ability to distinguish between two simple vowel sounds) predicts language development at 13, 16, and 24 months of age. These outcomes imply that phonetic perception plays a critical role in the early stages of language acquisition. They also suggest that measures of speech discrimination may facilitate the early detection of infants at risk of DLD. In fact, the phonetic abilities of children diagnosed with DLD have often been shown to lacking (Bishop & Edmunson, 1987). Usually, children with DLD perform significantly more poorly on speech perception tasks such as distinguishing between consonants in word pairs that differ by only one phoneme (Reed, 1989). Further evidence of a link between deficits in speech perception and language acquisition has been found in school-age children with DLD (Leonard et al., 1992; Stark & Heinz, 1996; Sussman, 2001). Thus, studies in which children with a variety of language impairments are compared with age-matched controls usually show that children with DLD display significant impairments in speech perception and phonetic/phonological representations. They also display more restricted lexical/semantic knowledge and vocabulary than their typically developing peers (Sheng & McGregor, 2010).

Children with DLD usually show poor performance than typically developing children in implicit tasks. For instance, Garraffa et al. (2015) showed that in a structural priming paradigm children with DLD exhibited impaired implicit learning mechanisms; indeed, this finding is already well-known in literature. In fact, through the semantic categorization task our aim was not to demonstrate that children with DLD show generalized implicit deficits. Here, we claimed that before testing children with DLD in their knowledge/representation of grammatical components (e.g., third-person direct object clitics, or word/non word repetition), it would be worth of investigation to use implicit semantic categorization tasks that do not require any production of language. In addition, as the children in our sample showed DLD in the expressive component mainly (i.e., more related to language-use), and that such deficits are usually detected in later development, we believe a language-free/implicit task is a better way to detect it earlier; albeit we know that children with DLD generally show poor performance in implicit tasks (when compared to same age peers).

The secondary aim of this study was to examine whether the effects of male gender bias and self-gender bias would follow a different pattern in children with DLD as compared to typically developing preschool children (Belacchi & Cubelli, 2012). Contrary to our hypothesis (i.e., that children with DLD would have greater difficulty in perceiving the difference between feminine and masculine in gender semantic categorization and that this would lessen gender-related effects), we observed similar effects of male gender bias and self-gender bias in both groups. More specifically, both groups of children displayed greater accuracy in categorizing masculine words than feminine ones (girls: *M* = 24.31 out of 32; boys: *M* = 24.11), thus confirming male gender bias in line with existing findings (e.g., Andonova et al., 2004; Belacchi & Cubelli, 2012). We found a significant interaction between children’s sex and noun gender; however, the self-bias effect was only observed in boys who were better at categorizing masculine nouns (than feminine nouns). The self-gender bias was not observed in girls, probably because the feminine grammatical gender is the marked gender and therefore, more difficult to learn, including for girls. Nonetheless, the girls in our sample were better at categorizing female nouns than were the boys, thus displaying greater sensitivity to animal nouns of their own biological sex (see Fig. 2). The absence of the self-gender bias effect could also be related to the fact that the sample was not perfectly gender-balanced (50 males vs. 20 females); there were more boys than girls because DLD is more frequently observed in males than in females (e.g., Leonard, 2014).

A limitation should be acknowledged. As previously described (see "Method" section), the groups of stimuli were not matched for word frequency and familiarity, since nouns of referential group were more frequent and more familiar than the stimuli belonging to the other categories (i.e., the epicenes). In addition, nouns from referential group are usually acquired earlier in development. However, as we noticed, the critical comparison is not between epicenes and referential, rather between different types of phonological and syntactic combinations; therefore, we believe this does not represent a shortcoming that reduces the validity of the task we presented here.

A further shortcoming should be recognized. As previously described, we were allowed to access the diagnosis but not the clinical records (with all tests administered and scores). However, we have to notice that our sample numerosity was very high (70 children with DLD) and thus participants selection was done a priori by the clinicians in charge of child evaluations; thus, we did not test directly all the children. On the contrary, in other studies with reduced numerosity, the authors could test each child individually administering a large series of tasks. This was not the case with our study, also because our aim was not to provide individual evaluation of children with DLD but to conduct an exploratory experimental study to show discriminant validity of the semantic categorization task, whose good psychometric properties were already shown by Belacchi and Artuso (2020).

It is also worth noticing that the Italian Consensus Conference did not produce clear-cut and univocal criteria to place a diagnosis of DLD but general recommendations only. Indeed, a specific indication of which measure is more reliable and predictive did not emerge. Therefore, it was recommended to use more measures to increase diagnostic accuracy. It also emerged the importance of early assessment, from 3 years onward (see also Rinaldi et al., 2021).

In future studies we plan to assess concurrent validity of the task comparing it to other traditional measures such as word/non word repetition task. In addition, it will be timely to collect data from samples with different DLD profiles to obtain further information about task discriminant validity.

In conclusion, assessing linguistic implicit competence in DLD children via an implicit grammatical gender semantic categorization task seems to be a promising approach to detecting linguistic impairments and informing ad hoc interventions. Such a task could also help to compare children with different DLD profiles and to refine our mapping of these profiles and the differences between them. Also, studies like this can inform the literature on grammatical gender representation and development (see Samuel et al., 2019). In addition, future research could focus on other developmental disorders with a language impairment component, such as, for example, intellectual disabilities or autism spectrum disorders, again with a view to advancing understanding of their specific cognitive-linguistic profiles.

## Data Availability

Data are available on request to the first/corresponding author.
